# ‘Why are we stuck in hospital?’ Understanding delayed hospital discharges for people with learning disabilities and/or autistic people in long‐stay hospitals in the UK

**DOI:** 10.1111/hsc.13964

**Published:** 2022-08-11

**Authors:** Rebecca Ince, Jon Glasby, Robin Miller, Anne‐Marie Glasby

**Affiliations:** ^1^ Department of Social Work and Social Care University of Birmingham Birmingham UK; ^2^ Changing Our Lives Crawley UK

**Keywords:** autism, delayed discharge, learning disabilities, long‐stay hospital, transforming care

## Abstract

Despite longstanding efforts at de‐institutionalisation, around 2000 people with learning disabilities and/or autistic people in England currently live in hospital settings, amidst reports of protracted stays, limited progress towards living more ordinary lives and scandals of abuse and poor care. Yet, there is relatively little research on why people with learning disabilities and/or autistic people are delayed in hospitals, and what exists has significant limitations. In particular, previous studies have rarely talked directly to people with learning disabilities and/or autistic people, their families and frontline staff about their experiences of living or working in such settings, the barriers to discharge and what would help more people to lead chosen lifestyles. This paper presents the findings of a structured literature review conducted between January and March 2021 on delayed discharges of people with learning disabilities in long‐stay hospital settings. It investigated: the proportion of people with learning disabilities delayed in long‐stay hospital settings, the suggested reasons for these delays and the proposed solutions. The literature reported delays for 11%–80% of inpatients in different settings. The reasons reported are related either to particular characteristics of the person (which we find problematic) or limitations of the system supporting them. However, delays were defined and reported inconsistently, reasons usually lacked depth and detail, and the majority of included studies did not engage directly with the people living in long‐stay settings, their families or frontline staff. Without listening to these voices, genuine solutions will be difficult to find.

**What is known about this topic**

- There are longstanding concerns about how long people with learning disabilities spend in hospital and the quality of their care.
- Many people in long‐stay hospitals may be ‘stuck’, that is clinically fit to be discharged, but unable to make this happen.
- Previous literature has identified issues such as the patient's level of need, funding and availability of suitable post‐hospital placements as potential reasons for delays.

**What this paper adds**

- Shows reported delayed discharges in different settings in the UK since 1990, ranging from 11% to 88% of inpatients.
- Uncovers the lack of voice of people using services, families and front‐line care staff in the existing literature.
- Identifies two types of reasons given for delays: relating to either the person themselves, or the wider system, but these lack detail and need to be explored further.

## 1 INTRODUCTION

While definitions and language vary, people with learning disabilities (sometimes known as people with ‘intellectual disabilities’, ‘developmental disorders’ or ‘learning impairments’, among other terms) are generally considered to have reduced cognitive or intellectual abilities and impaired social functioning, often requiring support to live independently (Department of Health, 2001, 2012). Enabling people with learning disabilities and also autistic people 1 to receive care and support at home rather than in potentially long‐stay hospital settings such as inpatient units, secure settings or assessment and treatment units (ATUs) has long been a key government priority. Internationally, there was a significant trend towards de‐institutionalisation over the 1970s–1990s, including trialling and scaling‐up models of specialised community‐based, non‐hospital support for people with learning disabilities, such as the intermediate care programme in the USA, the Trieste model in Italy and the Andover model in the UK, as well as the development of small group homes in Nordic countries (Mansell, 2006). More recently, there has also been increasing recognition of the particular needs of people with autism at a global level (WHO, 2022). The care mix in the UK varies between the four nations, but shares a peculiarly complex and multi‐sectoral makeup, with many categories of bed provision for different needs (Hatton, 2016), including services that are considered ‘community’ placements which strongly resemble institutions, as well as smaller hospitals that have a more ‘community’ feel, potentially blurring the distinction between types of provision.

A range of policies exist across the UK nations in response to reviews and incidents of poor care or scandal in services for people with learning disabilities. However, taking recent developments in England as an example (for illustrative purposes), the ‘Building the Right Support’ and ‘Transforming Care’ programmes were established after the Winterbourne abuse scandal was identified by a BBC TV documentary, ‘Panorama’ (Chapman, 2011). These aimed to enhance community capacity, thus reducing inappropriate hospital admissions and length of stay (NHS England and Partners, 2015a, 2015b). The overall goals were to reduce inpatient beds by 50%, enhance community services through 48 ‘Transforming Care Partnerships’ and ensure the use of independent ‘Care and Treatment Reviews’ (CTRs) for those in inpatient care. However, several targets were missed and significant challenges persist:

▶ In February 2015, NHS England and Partners (2015b, p. 6) committed to closing long‐stay institutions and discharging most patients, aiming for hospital care for 1300–1700 people by 2018. In 2019, Department of Health and Social Care (2019) set a further target of 400 additional discharges. But as of January 2021, 2040 people with learning disabilities were still hospital inpatients, 58% with a stay of over 2 years (NHS Digital, 2021).
▶ Various campaigning organisations (Duffy, 2019; Mencap, 2019; National Autistic Society, 2017; Voluntary Organisations Disability Group, 2018) have identified continuing issues with care in inpatient settings, including a lack of meaningful activity, abuse and inappropriate use of segregation and seclusion. In 2018 another undercover investigation found prolonged psychological and physical abuse at Whorlton Hall, a community provider (Plomin, 2019).
▶ Multiple official reviews have also been conducted, for example by the Parliamentary Joint Committee for Human Rights (2019) and the CQC (2020). There has also been criticism by Mencap (2019) of a lack of commitment to delayed discharges in the *NHS Long Term Plan* (NHS England, 2019), and in 2020 the Equality and Human Rights Commission announced a legal challenge to what it deemed a breach of the European Convention of Human Rights:
*Today we have launched a legal challenge against the Secretary of State for Health and Social Care over the repeated failure to move people with learning disabilities and autism into appropriate accommodation. We have longstanding concerns about the rights of more than 2000 people with learning disabilities and autism being detained in secure hospitals, often far away from home and for many years.* (Equality and Human Rights Commission, 2020)

In addition to issues around poor quality of life and mistreatment, hospital services are also very expensive, with average weekly costs of £3500 and annual costs of £180,000 per person (Mencap, 2019; National Audit Office, 2017), creating a negative cycle of channelling funds into hospital units instead of into the kind of community care that policies intended to create. Although this brief summary has focused on the specifics of English policies, similar issues exist across the UK nations, highlighted by the Bamford Review and Hospital Resettlement Programme in Northern Ireland (Palmer et al., 2014), the National Care Review conducted in Wales (Mills et al., 2020) and in ‘Coming Home: a review of out of area placements and delayed discharges for people with learning disabilities’ conducted by the Mental Welfare Commission for Scotland (MWCS, 2016). Across the UK, the goals of all these reviews and policies are laudable, but significant barriers to transferring people from restrictive settings remain, and we need a better collective understanding of what is standing in the way.

## 2 METHODS

To explore these issues, we conducted a narrative analytical review, summarising and interpreting the data presented in studies of different types to compare and contrast them in their original form (Mays et al., 2001). Its overall purpose was to identify the prevalence of delayed discharge for people with learning disabilities in long‐stay hospital settings, how this was measured, whether service users, families and staff had been included in the research, and the solutions proposed. To achieve this, we adopted an approach used in previous DH/NIHR research into delayed transfers of care (Glasby et al., 2006) and the appropriateness of emergency admissions (Thwaites et al., 2017), replicating a search previously published here in Health and Social Care in the Community.

The initial literature search was undertaken by a specialist health and social care library and literature searching team at the authors' institution. A range of health and social care databases were searched, selected on the basis of their relevance to the topic under investigation. These were:

- The Health Management Information Consortium database
- Medline
- The Social Science Citation Index
- The Applied Social Sciences Index and Abstracts
- Scopus
- Social Policy and Practice (including CareData, Social Care Online and AgeInfo)
- Social Services Abstracts

An additional search of the ‘grey’ literature (using the same terms as in the search of formal databases, via the search function of each website) via the websites listed below.

- Care Quality Commission
- Centre for Welfare Reform
- Challenging Behaviour Foundation
- Children's Commissioner for England
- Department for Health and Social Care
- Equality and Human Rights Commission
- Health and Social Care Scotland
- House of Commons/House of Lords Joint Committee on Human Rights
- Learning Disability England
- Learning Disability Wales
- Mencap
- Mental Welfare Commission for Scotland
- National Audit Office
- National Autistic Society
- NHS England
- Northern Ireland Assembly
- Northern Ireland Audit Office
- Scottish Commission for Learning Disability
- Scottish Government
- Scottish Learning Disability Observatory
- Social Care Wales
- Tizard Centre
- UK Parliament
- Voluntary Organisations Disability Group
- Welsh Audit Office
- Welsh Government
- Welsh Parliament

The search terms and operators used were selected to gather sources which covered the population (i.e. adults with learning disabilities and/or autism), as well as the correct care settings (i.e. long‐stay hospital provision for people with learning disabilities) and focusing on the specific issue under investigation (i.e. length of stay, delayed discharges or being ‘stuck’, rather than issues, treatments or processes unrelated to discharge). The search terms included as many variants and synonyms of “learning disabilities”, “delayed discharge” and “long‐term hospital” as possible, and Boolean operators were used to combine these (see Box 1 below for examples of search terms and Appendix S1 for the full list of search terms and operators used in each database search). The reference lists of articles included in this study were also searched for relevant titles.

BOX 1 **Sample search terms**

*Learning disabilities*—terms include: People with learning disabilities; Learning disability; Learning disabilities; Learning disorders; Learning difficulties; Intellectual disability; Intellectual development disorder; Mental disorders; Mental impairment; Developmental disabilities; Autism; Autism Spectrum Disorder; Child & adolescent mental health; Autistic spectrum; Language development disorder; Mental handicap.

*Long‐stay hospitals*—terms include: Long‐stay hospitals; Long stay patients; Mental health hospitals; Long stay patients; Long stay units; Secure settings; Secure units; Medium secure units; Forensic; Psychiatric secure units; Segregation; Secure accommodation; ATUs; Treatment facilities; Hospitalization/hospitalisation; Hospitals; Hospital units; Hospitals, special; Hospitals, psychiatric; NHS in‐patient; Child and adolescent mental health; CAMHS; Psychiatric units; Custodial institutions; Patient institutionalization; Assessment units; Inpatients; Institutionalization/institutionalization; Foreseeing psychiatric units; Hospital patients; In patients; Learning disability hospitals; Intellectual disability in patient units.

*Delayed discharge*—terms include: Delayed discharge; Delayed hospital discharge; Delayed transfer of care; Appropriateness of stay; Blocked beds; Hospital stay duration; Discharge planning; Patient discharge; Hospital discharge; Timely discharge; Treatment duration; Length of stay; Hospital patients; Bed availability; Patient transfer; Long term care; Bed availability; Future plan; Shift of care.

### 2.1 Inclusion and exclusion criteria

Each title and abstract generated by the initial search were reviewed independently by two members of the research team and selected for relevance to the overall aims and objectives of the study. Any articles found from the reference lists were included in this process. In the case of official data and reports (some of which tend to provide quarterly figures and updates), we included only the most recent official review of any national censuses from each of the four nations (rather than including every statistical bulletin in a broader series). Studies were included in the review if they met the following criteria:

- Reported original empirical data relating to the prevalence of or reasons for delayed discharges in UK‐based settings.
- Referred specifically to hospital or long‐term healthcare settings for people with learning disabilities and/or autistic people.
- Published from 1990 onwards (this year was chosen as it saw the passage of the UK's NHS and Community Care Act, which had a significant influence on community services available to those being discharged from hospital).

We consider long‐stay hospital settings to be specialist facilities registered as hospitals that are operated by either an NHS or independent sector provider, providing mental or behavioural healthcare in the UK for people with a learning disability or autism. This could be at any level of security (general/low/medium/high), and for people with any status under the Mental Health Act (i.e. admitted informally or detained). In defining ‘long‐stay hospital’ settings, we adapted the definition provided by NHS Digital (2021) (an official body which collates NHS data) in their regular statistical bulletins. While this refers to services in England, our definition includes services across the UK.

Studies that were excluded included: material published and/or based on data collected prior to 1990; local inspections where findings have been summarised in a national report; articles reporting findings from studies already included in the review; admission to non‐long stay settings; and the admission of people with mental health problems (unless the person has learning disabilities *and* mental health problems). Also excluded were studies which only described the hospital settings, characteristics of the hospital population, their treatment needs or evaluated the services and treatments on offer, without addressing length of stay, the discharge process or why delays might occur. Similarly, studies which solely reported patient experiences or long‐term outcomes *after* discharge were not included.

Included studies were summarised using criteria proposed by Mays et al. (2001) for assessing the quality of a range of studies. Specific data were identified and extracted from each paper on the following: the prevalence of delayed discharge; the methods used to ascertain this; whether the research explored the experiences of people with learning disabilities, their families or front‐line staff; the barriers to discharge; and any possible solutions identified.

## 3 RESULTS

### 3.1 Overview of papers

In total, the searches produced 785 potential studies, after de‐duplication from different databases (see Appendix S2 for the full search results). After review by two members of the team, only a very limited number of papers met the inclusion and exclusion criteria. Overall, there were 13 academic research articles included, of which one came from the reference list searches. Five national reviews from across the United Kingdom were also included:

1. England: A review of seclusion and restraint in hospitals for people with learning disabilities, carried out by the Care Quality Commission (CQC)—the regulator of health and care services in England. It explored the experiences and effects of long‐term hospital stays, segregation and seclusion, discharge and transition planning and barriers to people moving on (CQC, 2020).
2. Northern Ireland: A review of progress of the resettlement programme for delayed discharges, commissioned by the Northern Ireland Housing Executive who carried out the programme, also exploring reasons for slow progress (Palmer et al., 2014).
3. Scotland: A review of delayed discharges entitled ‘No Through Road’ conducted by the Mental Welfare Commission for Scotland, investigating the extent of and reasons for delayed discharges from learning disability hospital units across Scotland (MWCS, 2016).
4. Scotland: A review of all long stay, ‘out of area’ placements (people placed in services outside their local area), commissioned by the Scottish Government. It reports the extent and length of delays for out of area patients with learning disabilities and complex needs, and purported reasons for delays (MacDonald, 2018).
5. Wales: A National Care Review of the care and treatment of people with learning disabilities and/or autism in all 55 hospital units caring for Welsh citizens (Mills et al., 2020) which examined readiness for transition and the appropriateness of peoples' settings for their needs.

Of the 13 academic articles, 11 used bed census or retrospective case notes analysis and did not include qualitative data. Three of the 13 academic articles also tested a tool or protocol designed to reduce delayed discharges and only 5 interviewed stakeholders such as nurses, consultants or responsible clinicians. None of the academic articles included interviews with patients or families. The settings investigated across all studies ranged from open to secure wards, large hospitals, small rehabilitation units, ATUs, whole Trusts or single wards.

### 3.2 Prevalence of delayed discharge

The settings investigated by previous research varied enormously in size, type and scale (see Table 1 below), so where a rate or prevalence of delay was reported these are not necessarily comparable. Figures were often based on different definitions, or on proxy measures such as length of stay, readiness for discharge or the extent/presence of discharge plans (see below for further discussion). The range of delays reported are shown in Table 1 and range from less than 11% to over 80%.

**TABLE 1:** Prevalence of delayed discharge

Authors, date, country	Population/setting	Length of stay or delay (where included)	Prevalence of delayed discharge
Alexander et al. (2011) England	138 patients in a 64‐bed forensic service over a 6‐year period	The median length of stay for the discharged group was 2.8 years (1025 days) 75% of these stayed for less than 5 years	Of 61 patients who were still inpatients, 36 (59%) were considered ‘difficult to discharge long stay’ patients
Beer et al. (2005) England	200 inpatient across 20 low secure units (8 were for people with learning disabilities) in the South Thames region	Data not available	66 (33%) people were inappropriately placed; of these, 60 needed less security
CQC (2020) England	In depth reviews of 66 people as part of inspection visits to a wide range of mental health and learning disability services	Data not available	Discharge prevented due to lack of community services for 60% of the 66 people they met
Cumella et al. (1998) England	21 patients admitted for more than 3 months to an acute admissions facility in North Warwickshire	Mean length of stay beyond treatment needs estimated at approximately 6 months	18 out of 21 people (86%)
Devapriam et al. (2014) England	16‐bed specialist LD inpatient unit for people with learning disabilities	Data not available	29% (14 out of 49 people)
Dickinson and Singh (1991) England	Specialist “mental handicap hospital” in London	Average length of stay for ‘new long stay’ cohort was over 2 years	57 (55%) of 104 admissions were deemed ‘new long stay’ patients (resident for over 12 months)
Kumar and Agarwal (1996) England	“Mental handicap hospital” in south of England	Data not available	68.4% (188/275 people) considered suitable for discharge to a small home with minimal supervision; 72 (26%) suitable for discharge, but some difficulties in management likely
MacDonald (2018) Scotland	All but one Health and Social Care Partnerships in Scotland	More than 22% over 10 years; 9% for 5–10 years. Many people did not answer, but 13 people were delayed for 1 year+, and 10 people who were delayed had placements costing over £150,000 p.a. Only 51% had active discharge plans	67 people
MWCS (2016) Scotland	All 18 hospital units in Scotland—104 people's records (half of those in Scottish services)	50% over 3 years; just over 20% over 10 years	Nearly one‐third of current inpatients (32%) across Scotland were delayed discharges
Mills et al. (2020) Wales	256 patients with learning disabilities in units managed directly by, or commissioned by, NHS Wales (across 55 units)	Mean (all patients)—5.2 years current admission; 53% over 2 years; 19% over 10 years. 18% of current costs (5.994 million) could be reinvested in community services if all people who could be transitioned were transitioned	80 (54%) people could be considered for transition
Nawab and Findlay (2008) Scotland	Small 9 bed assessment and treatment unit in Lanarkshire	74% of all admissions = 1 week to 3 months; 20% = more than 3 months; 5% = more than a year	11% (18) considered delayed discharge
Oxley et al. (2013) England	2 small inpatient units (total of 12 beds) in London (1999–2001 vs. 2009–2011)	Mean length of stay: period 1 = 198.6 days (6 months); period 2 = 244.6 days (9 months)	67% (40/60) in period 1; 59% (24/41) in period 2
Palmer et al. (2014) Northern Ireland	All of Northern Ireland's learning disability hospital inpatient population, mostly at Muckamore Hospital Belfast	Average length of stay 6.2 years (includes short stays of days or weeks—so some must be very long)	No prevalence given but reported progress: 31 March 2014, 24 of 30 people from 2011 target list not resettled; March 2015: with new admissions, 49 people were delayed
Perera et al. (2009) Scotland	All 15 Health Boards in Scotland (range of settings)	Nearly half (47.9%) had been inpatients for more than 5 years	68 (17.52%) had delayed discharges
Taylor et al. (2017) England	Offenders with learning disabilities in an 18‐bed locked rehabilitation unit in Northeast England	See ‘prevalence of delayed discharge’ column for changes in length of stay	This is an evaluation of a discharge protocol, so no prevalence of delay given. However, the mean length of stay reduced by over 60 per cent from 39 months (3 years 3 months) to 14 months (1 year 2 months) during the project (implying a degree of delay). The rate of discharge was 7, 6 and 8 people over the first 3 years of the study, jumping to 16 discharges following use of the protocol (again implying previous delays)
Thomas et al. (2004) England	102 offenders with learning disabilities in all high security hospitals in England	Mean = 10.26 years; median = 8.5 years	32 (31%) did not need this level of security (different professionals disagreed on another 16 patients)
Washington et al. (2019) England	Two 21 bed Assessment and Treatment Units in North England	Mean admission length = 151 days	Just over 50% (36/70) experienced delayed discharge
Watts et al. (2000) England	Learning Disability Trust in Northeast England	At follow up 16 months later, 23 of the 44 patients identified as delays remained in hospital	44 (18%) out of 247 patients were delayed

The highest prevalence of delay was 86% or 18/21 patients reported by Cumella et al. (1998) in an acute admissions unit intended for shorter stays. Similarly, Oxley et al. (2013) and Washington et al. (2019) found almost 63% and over 50% of patients respectively were delayed in similar ATU settings. To clarify for readers not familiar with these service settings, some of this is expected as such services tend not to be designed for stays beyond a few months, but have often ended up with people resident for years, sometimes becoming de facto long‐stay settings due to delayed discharges. On the other hand, Nawab and Findlay (2008) reported only 11% of patients as being delayed. This was also an ATU, but here 74% of people stayed less than 3 months.

In studies of secure settings, delays were reported differently—often based on the appropriateness of the setting/level of security for the patients' needs. Delays were still very prevalent: 32% of patients in a low security unit needed less security (Beer et al., 2005) and similarly in a high secure setting around one third could be considered for transfer (Thomas et al., 2004). In the medium secure setting explored by Alexander et al. (2011), 50% of people were considered ‘difficult to discharge’—that is with a longer median length of stay than those discharged.

In those concerning general wards or a range of different service settings, delays were still significant, ranging from around 18% (Perera et al., 2009; Watts et al., 2000) to 29% (Devapriam et al., 2014) and 32% in one of the reviews conducted in Scotland (MWCS, 2016). In CQC's review across England, 60% of discharges were delayed:

*A lack of suitable care in the community prevented discharge for 60% of people we met. Most people in long‐term segregation needed bespoke packages of care in the community, but this was difficult to achieve.* (CQC, 2020, p. 29)

Those reporting proxies were higher: Kumar and Agarwal (1996) found 68.4% of people were considered ‘suitable for discharge’ (but still in hospital) and Mills et al. in their review across Wales found 54% of people ‘could be considered for transition’.

A small number of studies also report the extent of delays: MacDonald found 67 people in ‘out of area’ placements (i.e. not within the local authority where they lived) across Scotland were considered to have delayed discharges, one third of them for over a year. In Northern Ireland, Palmer et al. (2014) found that of 30 people identified as delayed discharges, only 6 were discharged between 2011 and 2014, leaving 24 people still in hospital, with 25 new admissions since 2011 who were also delayed. Devapriam et al. (2014) also noted the extent of delays at different stages of the discharge process (explored below), the majority being delayed for an average of 4 months (one patient over 2.5 years) at the first stage of assessment and identifying a suitable placement.

Throughout, there was little consistency in terminology and definitions of delayed discharge, making it impossible to meaningfully compare the extent of or reasons for delay between studies or to aggregate data. The majority of studies adopt either an explicit or an implicit definition that sees a ‘delayed discharge’ as occurring when a person remains in hospital after they have no clinical need to remain. However, studies in secure settings often focus on whether someone is ready to transfer to a less secure setting (remaining an in‐patient), and national reviews suggest some people are transferred to other hospitals (not really a ‘discharge’ in lay terms). Some studies use the terminology ‘difficult to discharge’ (Alexander et al., 2011), as well as assuming that lengths of stay exceeding a particular limit indicated a delay by default (Alexander et al., 2011; Dickinson & Singh, 1991; Washington et al., 2019, Watts et al., 2000). These varying interpretations generate important questions about subjectivity and perspective: in whose view is a person ready to move on? Who assesses whether the level of restriction is appropriate; what length of stay is excessive for different settings and on what basis (see below for further discussion)?

### 3.3 Length of stay

Length of stay is sometimes reported either as contextual information or as a proxy for delays. Some reported the proportion of stays for different lengths of time, others reported mean or median length of stay, and some a combination (Table 1). Oxley et al. (2013) also reported a longitudinal change in length of stay, with median stays increasing from 6 to 9 months across 4 years. Length of stay ranged significantly between settings—ATUs or similar had shorter lengths of stay than secure settings, ranging from weeks (Nawab & Findlay, 2008) to median stays of 3–6 months (Oxley et al., 2013; Washington et al., 2019). Notably, a large proportion of people stay in secure settings for many years: for example, 42% of people stayed over 5 years and 11% over 10 years in a medium secure setting (Alexander et al., 2011), mean lengths of stay in a locked rehabilitation unit were over 6 years (for those now discharged, Taylor et al., 2017) and mean lengths of stay reached over 10 years in a high secure setting (Thomas et al., 2004).

In studies reporting across a range of settings, often more than half of people were staying more than 5 years (Mills et al., 2020; Palmer et al., 2014; Perera et al., 2009). In Scotland, the MWCS's (2016) review across Scottish learning disability services similarly found around 70% of people staying longer than 3 years. Given these averages include a number of short‐stay settings, the figures indicate some very lengthy inpatient stays spanning decades.

### 3.4 ‘Explaining’ delayed discharge

The range of reasons given for delayed discharges are shown in Table 2 below, covering reasons associated with individual characteristics and reasons connected to the discharge process and wider system. This is largely similar to Glasby's (2003) review of delayed discharges from general hospitals, which explored individual, organisational and structural issues at stake, and argued for the need to work across multiple levels concurrently.

**TABLE 2:** Reasons cited for delayed discharge

Authors and date	Reasons for delayed discharge—characteristics	Reasons for delayed discharge—Process/system issues
Alexander et al. (2011)	More criminal sections and restriction orders; history of fire setting; having suffered abuse; diagnosis of personality disorder; history of substance misuse	Data not available
Beer et al. (2005)	Factors that might predict a delay were being young, being admitted on an informal basis, and not having ‘overactive’ as a reason for admission	May be knock‐on effects at different levels of security: *discharge problems at lower levels of security fail to free up low secure beds, creating discharge problems at higher levels of security* (p. 635)
CQC (2020)	Re‐traumatising and increased needs after failed community placements	Funding—availability, complexity and accessing, disputes over responsibility; commissioners' fears over high levels of risk and cost in community; lack of appropriate care in the community
Cumella et al. (1998)	One person's parents had left the country	Lack of places in suitable specialist accommodation or day care (13 people); funding disputes between NHS and local authority (4 people)
Devapriam et al. (2014)	Data not available	Awaiting assessment of future needs and identifying suitable placement—7 people (50%); awaiting social services funding or agreement—4 people; the remaining 3 people were delayed due no suitable placement available or legal issues
Dickinson and Singh (1991)	Psychiatric factors (increased previous admissions, family history and diagnosis of psychosis and dementia) and social factors (deceased parents and an inability to be discharged back to place of admission, particularly if admitted from home)	Data not available
Kumar and Agarwal (1996)	Of those suitable for discharge but who might be difficult to manage in the community, reported reasons/needs were: aggressive behaviour (24.5%); violent behaviour (8%); and self‐injury (6.4%)	Staff attitudes; previous experiences of the successes/failures of resettlement
MacDonald (2018)	Primarily male; 40% had mental health problems (most commonly bipolar disorder, anxiety, depression, schizophrenia); nearly 75% currently had challenging behaviour, over two thirds including physical aggression	Lack of accommodation (51%); lack of service providers (15%); other factors included legal/funding/geography issues
MWCS (2016)	Complex needs requiring specially commissioned service (e.g. 24/7 care with 1:1 or more staff); deterioration in the person's mental or physical health; needs escalate/incompatibility with other residents/placement becomes unsuitable	Funding (41%); housing (74%); no appropriate care provider (62%) (not mutually exclusive). Other reasons include lost places due to timing of available local authority funding with available appropriate placement; or delays in adaptations to properties, allocating a social worker, assessments, recruitment and training of support staff, and legal issues (e.g. guardianship)
Mills et al. (2020)	Data not available	Factors in readiness for transition include: professional judgement; patient's opinion; safety and risk to self and others; level of need and complexity etc
Nawab and Findlay (2008)	Data not available	Difficulty with placements—funding issues or lack of appropriate resources in the community (13/18); physical health—needing transfer to appropriate services (5/18); discharge and admissions protocols introduced—saw shorter stays and more discharges
Oxley et al. (2013)	Data not available	Lack of identification of suitable placement—69% of delayed discharges in 2009–2011 and 44% in 1999–2001
Palmer et al. (2014)	Data not available	Small number of new services and bed spaces created; lack of coordination between health, housing and social services; misalignment of funding streams; absence of an overall resettlement plan (e.g. monitoring, procurement); weak engagement by Trusts with patients and families; difficulty commissioning individual complex needs across health, social care and housing programme
Perera et al. (2009)	Data not available	47% (32)—due to social care reason (people awaiting assessment, or waiting for commissioning of services); 5%—due to healthcare reason; 47% (32)—no suitable facility available in the community/service development needed
Taylor et al. (2017)	Data not available	No reasons given but positive feedback on protocol suggests issues in: ‐ Clarity of process and roles, dedicated pre‐discharge planning meetings ‐ Partnership working—bringing departments together ‐ Risk management training for staff (particularly in community) ‐ Extra clinical support post‐discharge
Thomas et al. (2004)	Factors associated with continued need for high security: being younger, higher treatment and security needs, recent violent conduct and nature of initial offence	Majority of delays transferring to lower security were because a suitable placement did not seem to exist; the rest were due to funding issues, no bed available or not accepted (unsuitable services), or Home Office issues
Washington et al. (2019)	Individual characteristics acting as a barrier to discharge were only identified for 3% of delays (continuing mental [and physical health] difficulties)	For 83% of patients, delay was due to failure to source funding or find an alternative care provider. The remainder were delayed due to: placement/accommodation not ready; new trigger to mental health difficulties; finding a specialist bed; recruiting support staff to the provider
Watts et al. (2000)	Delayed patients tended to be older, admitted informally, having a more severe learning disability and a longer hospital stay. Those still delayed on follow up needed high levels of care (e.g. 24 h care, very experienced staff and high levels of staffing)	Lack of suitable accommodation (34 people); insufficient funding (10 people); carers unable to cope (17 people); insufficient clinical support (11 people); lack of suitable educational placement (13 people)

#### 3.4.1 Personal characteristics

Many of the studies reported reasons for delayed discharges or excessive lengths of stay through associations with particular characteristics of the person delayed (see Table 2), by trying to find statistical associations between length of stay or prevalence of delay and patient characteristics such as age, gender, behaviour, level of disability, co‐existing diagnoses and criminal record. For example, Washington et al. (2019) found that 61% of inpatients with ‘barriers to discharge’ had a secondary diagnosis of autism, while 41% had mental health diagnoses (e.g. bipolar, depression and anxiety). In general, a number of studies find challenging behaviour, psychiatric conditions and a higher degree of intellectual disability to be the main predictors of longer length of stay or difficulty discharging (Alexander et al., 2011; Beer et al., 2005; Dickinson & Singh, 1991; Kumar & Agarwal, 1996; MacDonald, 2018; Thomas et al., 2004; Washington et al., 2019, Watts et al., 2000). These were largely linked to risk and those perceived as higher risk to themselves or others were often described as more likely to be delayed, unsuitable for discharge, or not ready for a lower level of security. ‘Social’ factors such as a poor home environment or lack of home support were also mentioned (Dickinson & Singh, 1991), along with the patient having high physical care needs or ‘complex needs’ such as mobility issues, needing 24 h supervision, waking night staff or other intensive staffing needs (Kumar & Agarwal, 1996; MWCS, 2016; Thomas et al., 2004; Watts et al., 2000).

Interestingly Beer et al. (2005) and Watts et al. (2000) both found that being admitted informally (i.e. not detained under the Mental Health Act) was associated with being delayed or needing a higher level of security, suggesting that being detained under the Mental Health Act could be a positive factor in a timely discharge or transfer, possibly because detention automatically initiates a statutory process of regular care reviews and reassessment of the appropriateness of the setting.

However, focusing on individual characteristics feels problematic for various reasons. Firstly, some authors rightly recognise that each individual has a unique, complex set of characteristics and needs: the groups being studied were heterogenous and each individual had a particular biography (Alexander et al., 2011; Devapriam et al., 2014; Oxley et al., 2013; Watts et al., 2000). Therefore, basic demographics such as gender or age were rarely found to be useful predictors of longer lengths of stay or delays. As Oxley et al. (p. 38) observe:

It is important to keep in mind that individuals with intellectual disabilities accessing specialist inpatient services are more likely to present with complex clusters of symptoms and behavioural problems that may span several diagnostic categories.

Secondly, many studies report associations between characteristics, implying but not stating a causal relationship between the characteristics and the length of stay/delay. In some cases statistical analyses have been conducted on small samples, arguably making techniques such as regression analysis less useful for exploring reasons for delays than other approaches (see below for further discussion). Second, it can lead to over‐simplification: much of this literature ultimately concludes that working with people with multiple, complex needs is essentially complex—which is not a surprising finding. Finally, in the literature on older people delayed in general hospitals, there has been a concerted attempt to avoid labelling people as ‘bed blockers’, as this implies it is their fault. In practice, the vast majority of people would rather be at home, and the delay is due to system issues rather than any fault of the individual. In other areas of social policy, focusing on personal characteristics would be seen as ‘victim‐blaming’, and might be considered offensive.

#### 3.4.2 System issues

Many of the papers also give reasons for delays that are related to the process of discharge, such as administrative issues, funding and the availability of suitable placements. In most, these factors are identified from case notes and so vary significantly, often dependent on the local context and reporting categories used by specific services or staff at the time the notes were made. Some of the reasons given are also speculative rather than derived from data, and many lacked further explanation (e.g., a statement that there would be fewer delays if there were more suitable placements available in the community, without any real attempt to define what ‘suitable’ means, consider what kinds of placements are available/missing or reflect on whether more or different placements really would make a key difference—and no attempt to test any of this).

##### Lack of appropriate placement/services post‐discharge

A significant number of papers that explored reasons for delays report the main issue as there being no community placement available, or no appropriate placement for the person's needs. For example, in Thomas et al. (2004), both responsible medical officers and nurses in a secure unit believed the majority of delayed transfers were because alternative placements simply did not exist or beds were not available. Similarly, Watts et al. (2000), Nawab and Findlay (2008) and Cumella et al. (1998) all report more than 70% of people delayed due to a lack of suitable accommodation or day care, and Perera et al. (2009, p. 169) ascribe 47% of delays to there being no suitable facility available in the community. Similar themes also emerged from national reviews, with MWCS (2016) finding that 74% delays in Scotland were due to a lack of suitable housing and 64% due to a lack of suitable service provider. MacDonald (2018) similarly reported that 51% of those delayed and in hospitals out of area were due to a lack of accommodation, with 15% because of a lack of service providers (not just accommodation). In Northern Ireland, Palmer et al. (2014) found the low number of new community placements (termed ‘bed spaces’) was a factor in the slow progress made in discharging people.

However, it is sometimes difficult to know what this means: is it an absolute absence of placements, a lack of sufficiently specialised placements, a lack of fit between what providers can offer and what individuals need, and/or are the hospital‐based staff consulted in these studies simply not aware of what placements are possible in the community? For example, both Devapriam et al. (2014) and Oxley et al. (2013) reported that the majority of delays—50% and 69% respectively—were actually due to difficulties in identifying and/or securing a suitable placement rather than simply a lack of placements:

*Surprisingly, only one patient was delayed due to lack of availability of an appropriate placement in the community; the rest had existing community placements identified and only one other patient had to wait for a bespoke placement to be commissioned. This reiterates that the reason for delay in most cases is a system issue rather than a lack of available placements for complex care in the community.* (Devapriam et al., 2014, p. 213)

Where studies explored these issues in more detail, they pinpoint particular missing elements of community placements—for example, a lack of specialist staff, training or an inability to meet particularly complex patient needs (MacDonald, 2018; MWCS, 2016; Washington et al., 2019; Watts et al., 2000). For a small minority the reasons for delays included not being able to go back home or back to their original placement, either because the patients' needs changed and staff or family could no longer cope (Nawab & Findlay, 2008; Oxley et al., 2013), the placement had become unavailable (bed filled) or their family circumstances had changed, for example one patient's parents had died and another's were in another country (Dickinson & Singh, 1991). Together, a “lack of placement” seems to indicate all or some elements of a future placement being missing, whether that be related to family circumstances, housing, the level of care needed and the specialism/training of staff. In one sense, all delayed discharges are caused in part by the ‘lack of a suitable placement’, almost rendering this category so broadly defined that it loses all meaning.

##### Funding of patient care

The availability of public funding (whether this is the high cost of services, delays in seeking approval for funds to be spent or disagreements between different health and social care partners as to who funds the person's care) was the second most common reason for delays in transferring to lower security, according to Thomas et al. (2004). MWCS (2016) also found 41% of people were delayed due to ‘funding issues’, while for Watts et al. (2000) ‘insufficient funding’ contributed to 23% of delays. Funding issues obviously affect the availability and suitability of a placement and even where funding and placement issues have been reported separately, it is clear that these categories are not mutually exclusive, e with many patients delayed for both reasons (Cumella et al., 1998; Devapriam et al., 2014; Perera et al., 2009; Watts et al., 2000). Sometimes, agreeing funding seemed to be the issue (rather than necessarily the amount of money available), with Cumella et al. (1998) finding nearly a quarter of patients were delayed due to funding disputes between local authorities and (former) health authorities, and Devapriam et al. (2014) finding a similar proportion of people were awaiting funding decisions. Without giving statistics, CQC (2020) identified funding availability, disputes, access and complexity as major contributors to excessively long stays in hospital, and Palmer et al. (2014) noted significant difficulties in commissioning complex, individual care packages across health, social care and housing. As with labels such as ‘lack of suitable placements’, it is difficult to tell what delays due to ‘funding’ actually mean in practice. After all, people are often delayed in very expensive hospital settings, suggesting not an absence of funding but perhaps that existing funding is stuck in the wrong place in an inflexible system: difficulties moving funding creates difficulties moving people.

##### Discharge process issues

Broadly, the literature highlights two areas of the discharge process that seem particularly problematic—waiting for assessments and a lack of proactive discharge planning, often not using tools or protocols that are already available. Both Devapriam et al. (2014) and Perera et al. (2009) found around half of discharges were delayed whilst awaiting a social care assessment. Regarding discharge planning, Mills et al. (2020) reported that 82% of patients having no future placement identified, MacDonald (2018) found that around half of people in the Scottish services under review had no active discharge plans, and in England, the CQC (2020) found that 60% of people had no quality discharge plan in place. This indicates that problems for discharges can occur at multiple stages of the inpatient journey, including at the point of admission, which Devapriam et al. (2014) outlined as follows:

- Stage 1: Assessment of needs and identifying an appropriate placement.
- Stage 2: Awaiting funding decisions from Local Authority and Health Authority—including resolving disputes over responsibility.
- Stage 3: Awaiting authorisation of funding from the responsible authority.
- Stage 4: Waiting for package to be ready, for example staff trained, accommodation adapted.

Nearly half—and the largest proportion of patients—were delayed at the first stage for the longest period of time: an average of 4 months, but the longest reaching 2.5 years. MWCS (2016) also identified timing issues with the discharge process: for some patients, waiting for funding decisions at different stages resulted in potential placements being filled by someone else, indicating there were appropriate services but potentially not enough spaces in them, or a lack of mechanisms to prioritise people for transfer.

##### Changing service providers, policy and governance

Oxley et al. (2013), Devapriam et al. (2014), Mills et al. (2020), MacDonald (2018), MWCS (2016) and CQC (2020) also note a wider shift towards the use of private/independent providers in an increasingly multi‐sectoral mix of services. They suggest this influences delays for a number of reasons: concerns over the transparency of the offer, questions about quality and appropriateness of the care provided (particularly by private providers), and the intersection of multiple agencies and providers making coordination harder.

Naturally, there are challenges in governing a complex, multi‐sectoral system that directly impact discharge processes. In Northern Ireland, for example, Palmer et al. (2014) identified misalignments of funding streams and lack of coordination between health, housing, social services and social development departments to be a significant barrier to progress in discharging delayed patients. An overall resettlement plan including monitoring and procurement was also lacking, with weak engagement with patients and families by Health Trusts. The CQC (2020) also highlighted how disputes between local and national commissioners or between health and social care stakeholders can lead to a lack of agreement over responsibility for funding the person's care—especially during transition periods.

Governance issues also influence commissioning and CQC (2020) noted commissioners' fears as a barrier to developing community services, reporting that commissioners perceived higher risks in the community than hospitals with 24‐h care, and sometimes incorrectly assumed community packages are more expensive than hospital beds. Cumella et al. (1998) also found different commissioning approaches influenced the extent of delays, identifying three distinct approaches:

- A ‘devolved’ approach—local teams organise transition process and placements, commissioners approve funding.
- ‘No strategy’—reviewing patients' suitability for discharge/transfer case‐by‐case.
- The ‘clinical approach’—a resettlement officer liaises between providers and community teams throughout discharge process.

Of these, the third approach was identified as most successful in reducing delays, alongside specific discharge protocols and CTRs. This literature is from the late 1990s and refers to a period shortly after a significant effort at deinstitutionalisation, so relates less to recent policies and structures. However, the issues it uncovers suggest that—both now and historically—the roles, responsibilities and processes relating to discharging patients with learning disabilities from hospital have been poorly defined and coordinated across health and social care systems in the UK.

### 3.5 Perspectives and voices

Above all, a key argument of this paper is that the perspectives and voices of people using services, their families and front‐line care staff are often overlooked in the debate over delayed discharge (see Thwaites et al., 2017 for a similar argument with regards to older people in general hospitals). In our review, most of the data used derives from bed censuses, case notes and the views of the individual researchers (often a medical practitioner). Remarkably, *no* academic journal articles we included were able to assess the prevalence of delay, suggest reasons for those delays AND include the voices of service users, families and front‐line care staff. Whilst patient and family voices were entirely absent from the academic literature (see Table 3), they were sometimes present in the national reviews included (which were usually authored by or in collaboration with a third sector organisation or national health and social care body). Even professionals' voices (nurses, doctors, ward managers etc) were only found in five of the 13 academic papers included. These were included either to assess the appropriateness of the level of security for patients (Beer et al., 2005; Thomas et al., 2004), give further detail as to the reasons for delay (Cumella et al., 1998; Kumar & Agarwal, 1996) or, in one case, give feedback on a new discharge protocol (Taylor et al., 2017). However, these sometimes seemed like ‘add ons’ to the ‘main’ finding—the overall prevalence of delays (usually defined via bed census/case notes and based ultimately on the opinion of a lead researcher, usually a medic).

**TABLE 3:** Different perspectives included in previous research (or not)

Authors and date	Includes people using the services and/or families?	Includes front‐line staff and/or other professionals?
Alexander et al. (2011)	No	No
Beer et al. (2005)	No	Unit manager assessed ‘appropriateness of placement’ for each patient; data completed by a clinical lead who knew the patient
CQC (2020)	Yes—visited and spoke to patients and carers	Yes—frontline staff and commissioners interviewed; questionnaires completed by service managers
Cumella et al. (1998)	No	Yes—nurses, consultants and staff responsible for purchasing learning disability services
Devapriam et al. (2014)	No	No
Dickinson and Singh (1991)	No	No
Kumar and Agarwal (1996)	No	Yes—nurses in charge of each ward completed the questionnaire, usually charge nurse or ward sister
MacDonald (2018)	Yes—individual case studies supplied by Partnerships and by family carers	Yes—meetings with health and social care providers and with Health and Social Care Partnerships
MWCS (2016)	Yes—spoke to individual patients, involved carers via meetings and questionnaires	Yes—questionnaires to clinical service managers and nurses, spoke to nurses
Mills et al. (2020)	Yes—advocates worked with 17 patients directly	Yes—practitioners (multiple, including therapy staff, nursing team)
Nawab and Findlay (2008)	No	No
Oxley et al. (2013)	No	No
Palmer et al. (2014)	Sister report on patient experiences of resettlement includes service users and carers	Consultations with policymakers, programme planners, service commissioners and senior manager
Perera et al. (2009)	No	No
Taylor et al. (2017)	No	13 stakeholders (commissioners, nursing staff, clinicians, care staff, social workers etc) gave feedback on protocol
Thomas et al. (2004)	No	Responsible medical officers and primary nurses identified the appropriateness of security level for each patient
Washington et al. (2019)	No	No
Watts et al. (2000)	No	No

In contrast, the national reviews included from across the UK tried to include perspectives from a range of stakeholders—service users, carers, frontline staff, managers and commissioners. They did this using a range of methods such as questionnaires, focus groups, observations and interviews designed to delve deeper into the experiences and quality of care and practices involved, and the reasons behind delays. For example, Mills et al. (2020) included multiple perspectives at each visit:

*Information was gathered, during site visits to each unit, from the patient, therapy staff, nursing team, clinical notes and prescription charts. It was not possible to have a discussion with the patients' families and carers ….* (Mills et al., 2020, p. 21)

Palmer et al. (2014) also sought views on the effectiveness of the policy programme overall, using:

*…consultations with policymakers, programme planners, service commissioners and senior managers involved in resettlement, and in the delivery of housing and support services to resettled people, to explore their views and perceptions of: the pace of and influences on the rate of resettlement; standards and issues in the provision of housing, care and support services; views about the aims of the resettlement programme and the extent to which they have been or are being achieved.* (Palmer et al., 2014, p. 8)

### 3.6 Recommendations and implications for practice

Generally, the recommendations made fall into three broad types. Firstly, several studies stress the underlying principles of better provision, such as more and better services in the community for people with learning disabilities and/or autistic people (Beer et al., 2005; Cumella et al., 1998; Dickinson & Singh, 1991; Kumar & Agarwal, 1996; Thomas et al., 2004). Many also see closer joint working and coordination of services between social services and the NHS as a priority (CQC, 2020; Devapriam et al., 2014; Mills et al., 2020; Nawab & Findlay, 2008; Oxley et al., 2013), including suggestions such as joint development of a greater range of community services or packages of care for complex needs (CQC, 2020; MacDonald, 2018). Secondly, studies make recommendations in terms of knowledge and information, both relation to services and to research—building understanding, gathering and reporting data and monitoring progress. Finally, there are specific recommendations for changes to the management and delivery of services for people with learning disabilities, and specific calls for improved discharge processes. Almost all of the papers included call for more high‐quality research—some specifically for studies comparing different sites, settings and approaches rather than studies of singular sites or interventions (Alexander et al., 2011; Taylor et al., 2017). Only one paper (Taylor et al., 2017) specifically recommends more focus on service user and family experiences and perspectives. In relation to services or the system, MWCS (2016), Perera et al. (2009) and CQC (2020) suggest a standard reporting and monitoring system for delayed discharges, including reasons for delays (and admissions), whether or not reviews have taken place and protocols been followed. Alexander et al. (2011) also recommend outcomes‐based commissioning in order to capture the complexity of people's needs and perhaps avoid the largely useless exercise of trying to explain delays using individual characteristics as described above.

Many that include recommendations about the discharge process itself call for more streamlined processes, earlier and better discharge planning with greater involvement of service users and families (CQC, 2020; Cumella et al., 1998; Devapriam et al., 2014; Nawab & Findlay, 2008) and consistent use of available tools, protocols and legal frameworks such as CTRs, the Care Programme Approach, the Mental Health Act and existing discharge protocols (Cumella et al., 1998; Mills et al., 2020; Nawab & Findlay, 2008; Watts et al., 2000). This includes one study calling for greater use of a specific decision making tool for addressing delayed discharges (Devapriam et al., 2014).

Other recommendations relate to responsibilities, governance and relationships between stakeholders at different levels, ranging from suggesting a national commissioner responsible for reducing delayed discharges (CQC, 2020) to a designated professional within local services whose remit is to manage and streamline discharges, like the resettlement officer or responsible person role proposed by Cumella et al. (1998) and Devapriam et al. (2014) respectively. Linking to the purported lack of suitable placements in the community, some recommendations (but surprisingly few) champion changes to existing community provision. For example, Washington et al. (2019) focussed on specific skills training for those working in the community, in supporting people with a combination of learning disabilities or autism, mental health needs and challenging behaviour. MWCS (2016) call for specific training in positive behaviour support (PBS), specialist support for co‐existing autism and specific support for families and carers in times of crisis, located in the community. Others call for dedicated rehabilitation spaces during any transition (Cumella et al., 1998; Taylor et al., 2017), or models which seek to reduce risk and readmissions by continuing clinical support from the current hospital team during and after the move to the new setting (Oxley et al., 2013; Washington et al., 2019).

## 4 DISCUSSION

This review has explored the extent of delayed discharges for people with learning disabilities from long‐stay hospitals across the UK, the reported reasons behind these delays, the range of recommendations made to address the problem and the extent to which service users, families and front‐line care staff have been engaged in previous research. We found that a very significant proportion of people across various long‐stay settings are considered to be delayed or experiencing excessively long stays—some for decades. The reasons for this are broadly reported to be because of the extent or complexity of the individual's needs, or because of system issues such as a lack of suitable services in the community, disputes and issues with funding, poorly designed or implemented discharge or transfer processes, and wider problems with governance, commissioning and inter‐agency relationships. However, the use of statistical analysis to link particular individual characteristics with delays or longer stays was generally unhelpful and lacked explanatory detail, running the risk of ‘blaming the victim’. Explanations such as ‘funding’ or ‘lack of suitable placements’ provide some sense of what might help, but often lack detail and may over‐simplify more complex realities. Moreover, the range of solutions proposed to improve the situation around delayed discharges often appear overly generalised, such as calls for more development of specialist community services and clarity over who has political and financial responsibility for the problem, issues which have already been highlighted in decades of UK policy programmes.

### 4.1 Limitations

A very limited number of articles met the inclusion criteria, and the lack of inclusion of patients and family members' voices in the academic studies included is notable. Considering this is a high‐profile, long‐term and hotly debated issue, it appears to be significantly under‐researched, with existing claims to knowledge limited to a handful of very context‐specific/professionally‐dominated studies and national reviews in response to particular controversies. An additional limitation is that delayed discharge (or ‘being stuck’ in hospital) is defined and reported so inconsistently across the UK, resulting in such varied terminology that meaningful comparisons of rates of delayed discharges across different studies and locations are very difficult. The lack of patient and family involvement in the academic research studies could relate to the complex methodological and ethical considerations needed to work more closely with this population in research, (which academics may find prohibitive when seeking undertaking research in this area), or it could be that there is a philosophical divide between quantitative and qualitative methods: very few studies assessed both the prevalence of delayed discharges AND directly gathered qualitative data on the experiences of the people involved. In particular, it could be that—as a society—we do not value the lived experience of people who draw on care and support and their families—as a source of insight and expertise in its own right. Either way, these perspectives are the most notable absence in the literature and this inevitably results (at best) in a partial picture of why people are stuck in hospital and what might make a difference.

## 5 CONCLUSION

Above all else, any further research in this area must include the lived experience of people living in long‐stay hospitals and their families, as well as the practice knowledge of front‐line staff. Such perspectives represent a key form of expertise that we neglect at our peril, and it is difficult to see how we might produce genuine solutions to these longstanding issues without drawing more fully on these insights. Linked to this, there is a need to move beyond broadbrush explanations (‘lack of suitable placements’ etc) to unpick what this actually means, understand what might be needed to resolve the perceived issue and actually put such proposed measures in place. Future research and policy should also adopt standardised definitions, as is the case in other service settings (general hospital care for older people, for example). Proxy indicators of delayed discharge such as length of stay or number of people with discharge plans, coupled with a general lack of precision in terms of definitions, mean that data cannot be aggregated and that the extent of the issue cannot be fully understood. Beyond the prevalence of delay, there is also insufficient understanding of the amount of time different people are delayed, what this feels like and the impact it has on subsequent outcomes. Despite widespread and longstanding official commitment to enabling people with learning disabilities and/or autistic people to come out of long‐stay hospitals and lead more ordinary lives in the community, too many people are still ‘stuck’ in hospital—and it is nothing short of a national scandal that we still do not know enough about why this is or what would genuinely make a difference.

## AUTHOR CONTRIBUTIONS

Rebecca Ince: Writing – original draft (lead); investigation; writing – review and editing (equal); Jon Glasby: Conceptualization (equal); methodology (lead); writing – original draft (supporting); writing – review and editing (equal). Anne‐Marie Glasby: Conceptualization (equal); investigation; writing – review and editing (equal). Robin Miller: Conceptualization (equal); Writing: review and editing (equal).

## CONFLICT OF INTEREST

There are no conflicts of interest arising from this article.

## Supporting information


Appendix S1

Appendix S2

## Data Availability

Data sharing is not applicable to this article as no new data were created or analyzed in this study.

## Endnote

1 NB For the remainder of this article, we use the term ‘people with learning disabilities’ as a shorthand for this broader term.
