# Supplementary material for: ‘Why are we stuck in hospital?’ Understanding delayed hospital discharges for people with learning disabilities and/or autistic people in long‐stay hospitals in the UK
Source: Health Soc Care Community. 2022 Aug 11;30(6):e3477–92. doi: 10.1111/hsc.13964 (PMC10087420; doi:10.1111/hsc.13964)
Supplement: Supplementary file 1 — Appendix S1 [file HSC-30-e3477-s002.docx]

Appendix A

All search terms used for each database

| **Database** | **Search Strategy** |
| --- | --- |
| **HMIC** | Database: HMIC Health Management Information Consortium <1979 to November 2020> Search Strategy:  --------------------------------------------------------------------------------  1 exp Learning disabilities/ (6552)  2 exp Learning disorders/ (226)  3 exp autism/ (522)  4 exp autistic spectrum disorders/ (590)  5 "people with learning disabilit$".ti,ab. (1471)  6 learning disabilit$.ti,ab. (2818)  7 learning disorder$.ti,ab. (4)  8 learning difficult$.ti,ab. (1360)  9 intellectual disabilit$.ti,ab. (330)  10 intellectual development disorder$.ti,ab. (0)  11 developmental disabilit$.ti,ab. (60)  12 autism$.ti,ab. (502)  13 "child & adolescent mental health".ti,ab. (15)  14 autistic spectrum$.ti,ab. (93)  15 language development disorder$.ti,ab. (0)  16 mental handicap$.ti,ab. (1220)  17 1 or 2 or 3 or 4 or 5 or 6 or 7 or 8 or 9 or 10 or 11 or 12 or 13 or 14 or 15 or 16 (8354)  18 exp Long stay hospitals/ (71)  19 exp Long stay patients/ (289)  20 exp Long stay units/ (44)  21 exp Secure units/ (434)  22 exp Medium secure units/ (63)  23 exp Segregation/ (22)  24 exp Secure accommodation/ (590)  25 exp Treatment facilities/ (685)  26 exp Hospitalisation/ (7091)  27 exp hospitals/ (15432)  28 exp psychiatric units/ (278)  29 exp Custodial institutions/ (2016)  30 exp psychiatric secure units/ (140)  31 exp Secure accommodation/ (590)  32 exp Patient institutionalisation/ (19)  33 exp Assessment units/ (37)  34 exp Special hospitals/ (322)  35 exp Mental health hospitals/ (947)  36 "inpatient$".ti,ab. (3752)  37 (institutionalisation$ or institutionalization$).ti,ab. (252)  38 long stay hospital$.ti,ab. (253)  39 long stay unit$.ti,ab. (11)  40 long stay patient$.ti,ab. (202)  41 secure setting$.ti,ab. (43)  42 secure unit$.ti,ab. (468)  43 medium secure unit$.ti,ab. (78)  44 segregat$.ti,ab. (224)  45 secure accomodat$.ti,ab. (6)  46 atu$.ti,ab. (10)  47 "assessment and treatment unit$".ti,ab. (11)  48 treatment facilit$.ti,ab. (101)  49 hospitali?ation.ti,ab. (1503)  50 hospital$.ti,ab. (50036)  51 nhs in-patient$.ti,ab. (5)  52 in-patient$.ti,ab. (6504)  53 camh$.ti,ab. (225)  54 psychiatric unit$.ti,ab. (273)  55 custodial institution$.ti,ab. (4)  56 psychiatric secure unit$.ti,ab. (1)  57 secure accomodat$.ti,ab. (6)  58 patient institutional$.ti,ab. (1)  59 assessment unit$.ti,ab. (142)  60 special hospital$.ti,ab. (258)  61 psychiatric hospital$.ti,ab. (945)  62 mental health hospital$.ti,ab. (54)  63 foreseeing psychiatric unit$.ti,ab. (0)  64 exp Hospital patients/ (2450)  65 exp In patients/ (1593)  66 or/18-65 (66334)  67 exp Delayed discharge/ (270)  68 exp Patient discharge/ (2090)  69 exp Blocked beds/ (159)  70 exp hospital stay duration/ (794)  71 exp Discharge planning/ (336)  72 exp Hospital patients/ (2450)  73 exp Bed availability/ (158)  74 exp Patient transfer/ (377)  75 exp Long term care/ (1864)  76 delayed discharge$.ti,ab. (267)  77 delayed hospital discharge$.ti,ab. (27)  78 delayed transfer of care$.ti,ab. (4)  79 appropriateness of stay$.ti,ab. (0)  80 blocked bed$.ti,ab. (13)  81 hospital stay duration$.ti,ab. (4)  82 discharge plan$.ti,ab. (388)  83 patient discharge$.ti,ab. (148)  84 hospital discharge$.ti,ab. (820)  85 timely discharge$.ti,ab. (20)  86 treatment duration$.ti,ab. (47)  87 length of stay$.ti,ab. (1528)  88 bed avail$.ti,ab. (91)  89 patient transfer$.ti,ab. (63)  90 long term care$.ti,ab. (1959)  91 long stay care$.ti,ab. (97)  92 future plan$.ti,ab. (317)  93 shift of care$.ti,ab. (56)  94 or/67-93 (10381)  95 17 and 66 and 94 (152)  96 exp Learning disability hospitals/ (153)  97 learning disability hospital$.ti,ab. (8)  98 intellectual disability in-patient unit$.ti,ab. (1)  99 96 or 97 or 98 (161)  100 94 and 99 (16)  101 95 or 100 (155)  102 from 101 keep 1-113 (113) |
| **Social Policy & Practice** | Database: Social Policy and Practice <202010> Search Strategy:  --------------------------------------------------------------------------------  1 learning disabilities.mp. [mp=abstract, title, publication type, heading word, accession number] (14298)  2 learning disorders.mp. [mp=abstract, title, publication type, heading word, accession number] (41)  3 autism.mp. [mp=abstract, title, publication type, heading word, accession number] (1859)  4 autism spectrum disorder.mp. [mp=abstract, title, publication type, heading word, accession number] (252)  5 "people with learning disabilit$".ti,ab. (3518)  6 learning disabilit$.ti,ab. (6676)  7 learning disorder$.ti,ab. (23)  8 learning difficult$.ti,ab. (2842)  9 intellectual disabilit$.ti,ab. (2731)  10 intellectual development disorder$.ti,ab. (0)  11 developmental disabilit$.ti,ab. (495)  12 autism$.ti,ab. (1602)  13 "child & adolescent mental health".ti,ab. (7)  14 autistic spectrum$.ti,ab. (458)  15 language development disorder$.ti,ab. (0)  16 mental handicap$.ti,ab. (500)  17 1 or 2 or 3 or 4 or 5 or 6 or 7 or 8 or 9 or 10 or 11 or 12 or 13 or 14 or 15 or 16 (16935)  18 long stay hospitals.mp. [mp=abstract, title, publication type, heading word, accession number] (104)  19 long stay patients.mp. [mp=abstract, title, publication type, heading word, accession number] (67)  20 long stay units.mp. [mp=abstract, title, publication type, heading word, accession number] (0)  21 secure units.mp. [mp=abstract, title, publication type, heading word, accession number] (564)  22 medium secure units.mp. [mp=abstract, title, publication type, heading word, accession number] (190)  23 segregation.mp. [mp=abstract, title, publication type, heading word, accession number] (869)  24 secure accomodation.mp. [mp=abstract, title, publication type, heading word, accession number] (2)  25 treatment facilities.mp. [mp=abstract, title, publication type, heading word, accession number] (85)  26 hospitalisation.mp. [mp=abstract, title, publication type, heading word, accession number] (505)  27 hospitals.mp. [mp=abstract, title, publication type, heading word, accession number] (6877)  28 psychiatric units.mp. [mp=abstract, title, publication type, heading word, accession number] (216)  29 custodial institutions.mp. [mp=abstract, title, publication type, heading word, accession number] (399)  30 psychiatric secure units.mp. [mp=abstract, title, publication type, heading word, accession number] (0)  31 patient institutionalisation.mp. [mp=abstract, title, publication type, heading word, accession number] (1)  32 assessment units.mp. [mp=abstract, title, publication type, heading word, accession number] (17)  33 special hospitals.mp. [mp=abstract, title, publication type, heading word, accession number] (57)  34 mental health hospitals.mp. [mp=abstract, title, publication type, heading word, accession number] (33)  35 "inpatient$".ti,ab. (1565)  36 (institutionalisation$ or institutionalization$).ti,ab. (615)  37 long stay hospital$.ti,ab. (208)  38 long stay unit$.ti,ab. (3)  39 long stay patient$.ti,ab. (71)  40 secure setting$.ti,ab. (150)  41 secure unit$.ti,ab. (376)  42 medium secure unit$.ti,ab. (133)  43 segregat$.ti,ab. (972)  44 secure accomodat$.ti,ab. (2)  45 atu$.ti,ab. (11)  46 "assessment and treatment unit$".ti,ab. (41)  47 treatment facilit$.ti,ab. (144)  48 hospitali?ation.ti,ab. (656)  49 hospital$.ti,ab. (12321)  50 nhs in-patient$.ti,ab. (3)  51 in-patient$.ti,ab. (1707)  52 camh$.ti,ab. (513)  53 psychiatric unit$.ti,ab. (165)  54 custodial institution$.ti,ab. (24)  55 psychiatric secure unit$.ti,ab. (1)  56 secure accomodat$.ti,ab. (2)  57 patient institutional$.ti,ab. (1)  58 assessment unit$.ti,ab. (50)  59 special hospital$.ti,ab. (91)  60 psychiatric hospital$.ti,ab. (545)  61 mental health hospital$.ti,ab. (56)  62 foreseeing psychiatric unit$.ti,ab. (0)  63 hospital patients.mp. [mp=abstract, title, publication type, heading word, accession number] (165)  64 in patients.mp. [mp=abstract, title, publication type, heading word, accession number] (1455)  65 or/18-64 (18874)  66 delayed discharge.mp. [mp=abstract, title, publication type, heading word, accession number] (296)  67 patient discharge.mp. [mp=abstract, title, publication type, heading word, accession number] (72)  68 blocked beds.mp. [mp=abstract, title, publication type, heading word, accession number] (11)  69 hospital stay duration.mp. [mp=abstract, title, publication type, heading word, accession number] (0)  70 discharge planning.mp. [mp=abstract, title, publication type, heading word, accession number] (363)  71 hospital patients.mp. [mp=abstract, title, publication type, heading word, accession number] (165)  72 bed availability.mp. [mp=abstract, title, publication type, heading word, accession number] (77)  73 patient transfer.mp. [mp=abstract, title, publication type, heading word, accession number] (5)  74 long term care.mp. [mp=abstract, title, publication type, heading word, accession number] (5252)  75 delayed discharge$.ti,ab. (268)  76 delayed hospital discharge$.ti,ab. (33)  77 delayed transfer of care$.ti,ab. (16)  78 appropriateness of stay$.ti,ab. (0)  79 blocked bed$.ti,ab. (11)  80 hospital stay duration$.ti,ab. (1)  81 discharge plan$.ti,ab. (287)  82 patient discharge$.ti,ab. (33)  83 hospital discharge$.ti,ab. (637)  84 timely discharge$.ti,ab. (31)  85 treatment duration$.ti,ab. (15)  86 length of stay$.ti,ab. (437)  87 bed avail$.ti,ab. (13)  88 patient transfer$.ti,ab. (6)  89 long term care$.ti,ab. (3997)  90 long stay care$.ti,ab. (69)  91 future plan$.ti,ab. (403)  92 shift of care$.ti,ab. (30)  93 or/66-92 (7594)  94 17 and 65 and 93 (122)  95 learning disability hospitals.mp. [mp=abstract, title, publication type, heading word, accession number] (11)  96 learning disability hospital$.ti,ab. (20)  97 intellectual disability in-patient unit$.ti,ab. (0)  98 95 or 96 or 97 (20)  99 93 and 98 (4)  100 94 or 99 (122)  101 limit 100 to yr="1990 -Current" (118)  102 united kingdom.mp. [mp=abstract, title, publication type, heading word, accession number] (88672)  103 great britain.mp. [mp=abstract, title, publication type, heading word, accession number] (2019)  104 england.mp. [mp=abstract, title, publication type, heading word, accession number] (40100)  105 northern ireland.mp. [mp=abstract, title, publication type, heading word, accession number] (4294)  106 scotland.mp. [mp=abstract, title, publication type, heading word, accession number] (18663)  107 wales.mp. [mp=abstract, title, publication type, heading word, accession number] (12905)  108 UK.mp. [mp=abstract, title, publication type, heading word, accession number] (23480)  109 102 or 103 or 104 or 105 or 106 or 107 or 108 (134052)  110 101 and 109 (59) |
| **Medline** | Database: Ovid MEDLINE(R) <1946 to January Week 5 2021> Search Strategy:  --------------------------------------------------------------------------------  1 exp Learning Disabilities/ (22262)  2 exp Autistic Disorder/ (20784)  3 "people with learning disabilit$".ti,ab. (594)  4 learning disabilit$.ti,ab. (7085)  5 learning disorder$.ti,ab. (1048)  6 learning difficult$.ti,ab. (2080)  7 intellectual disabilit$.ti,ab. (14282)  8 intellectual development disorder$.ti,ab. (12)  9 developmental disabilit$.ti,ab. (4714)  10 autism$.ti,ab. (36447)  11 "child & adolescent mental health".ti,ab. (18)  12 autistic spectrum$.ti,ab. (1461)  13 language development disorder$.ti,ab. (44)  14 mental handicap$.ti,ab. (1131)  15 *"Intellectual Disability"/ (39112)  16 "Developmental Disabilities"/ (20546)  17 exp Language Development Disorders/ (6561)  18 1 or 2 or 3 or 4 or 5 or 6 or 7 or 8 or 9 or 10 or 11 or 12 or 13 or 14 or 15 or 16 or 17 (129710)  19 exp Hospitalization/ (250167)  20 exp Hospitals/ (280639)  21 exp Hospitals, Psychiatric/ (25372)  22 exp Hospitals, Special/ (64867)  23 exp Inpatients/ (23027)  24 "inpatient$".ti,ab. (96476)  25 (institutionalisation$ or institutionalization$).ti,ab. (4392)  26 long stay hospital$.ti,ab. (212)  27 long stay unit$.ti,ab. (36)  28 long stay patient$.ti,ab. (464)  29 secure setting$.ti,ab. (101)  30 secure unit$.ti,ab. (220)  31 medium secure unit$.ti,ab. (75)  32 segregat$.ti,ab. (62769)  33 secure accomodat$.ti,ab. (0)  34 atu$.ti,ab. (1299)  35 "assessment and treatment unit$".ti,ab. (10)  36 treatment facilit$.ti,ab. (4616)  37 hospitali?ation.ti,ab. (123210)  38 hospital$.ti,ab. (1138004)  39 nhs in-patient$.ti,ab. (7)  40 in-patient$.ti,ab. (1540155)  41 camh$.ti,ab. (449)  42 psychiatric unit$.ti,ab. (2466)  43 custodial institution$.ti,ab. (43)  44 psychiatric secure unit$.ti,ab. (1)  45 secure accomodat$.ti,ab. (0)  46 patient institutional$.ti,ab. (54)  47 assessment unit$.ti,ab. (574)  48 special hospital$.ti,ab. (365)  49 psychiatric hospital$.ti,ab. (10893)  50 mental health hospital$.ti,ab. (281)  51 foreseeing psychiatric unit$.ti,ab. (0)  52 or/19-51 (2767212)  53 exp Patient Discharge/ (31215)  54 exp "Length of Stay"/ (91318)  55 exp Bed Occupancy/ (2552)  56 exp Patient Transfer/ (8732)  57 exp Long-Term Care/ (26341)  58 delayed discharge$.ti,ab. (416)  59 delayed hospital discharge$.ti,ab. (65)  60 delayed transfer of care$.ti,ab. (2)  61 appropriateness of stay$.ti,ab. (8)  62 blocked bed$.ti,ab. (19)  63 hospital stay duration$.ti,ab. (386)  64 discharge plan$.ti,ab. (3288)  65 patient discharge$.ti,ab. (1771)  66 hospital discharge$.ti,ab. (26242)  67 timely discharge$.ti,ab. (112)  68 treatment duration$.ti,ab. (9923)  69 length of stay$.ti,ab. (50186)  70 bed avail$.ti,ab. (312)  71 patient transfer$.ti,ab. (1279)  72 long term care$.ti,ab. (18937)  73 long stay care$.ti,ab. (96)  74 future plan$.ti,ab. (2409)  75 shift of care$.ti,ab. (202)  76 or/53-75 (214143)  77 18 and 52 and 76 (605)  78 learning disability hospital$.ti,ab. (4)  79 76 and 78 (1)  80 77 or 79 (605)  81 exp United Kingdom/ (370163)  82 exp England/ (107882)  83 exp Northern Ireland/ (4938)  84 exp Scotland/ (25020)  85 exp Wales/ (14322)  86 (england or northern ireland or scotland or wales).mp. (165572)  87 (United Kingdom or Great Britain or UK).mp. (303171)  88 81 or 82 or 83 or 84 or 85 or 86 or 87 (462027)  89 80 and 88 (85)  90 limit 89 to yr="1990 -Current" (64) |
| **SSCI*** | (("learning disabilit*" or "learning disorder*" or "learning difficult*" or autism or "autistic spectrum disorder*" or "people with learning disability*" or "intellectual disabilit*" or "intellectual development disorder*" or "child & adolescent mental health" or "autistic spectrum*" or "language development disorder*" or "mental handicap*"))   AND TOPIC: (("Long stay hospital*" or "Long stay patient*" or "long stay unit*" or "secure unit*" or "medium secure unit*" or segregate* or "treatment facilit*" or hospitali?ation or hospital* or "psychiatric unit*" or "custodial institution*" or "psychiatric secure unit*" or "secure accommodat*" or "patient institutional*" or "assessment unit*" or "special hospital*" or "mental health hospital*" or "inpatient*" or institutionalisation* or institutionalization* or "secure setting*" or atu* or "assessment and treatment unit*" or "nhs in-patient*" or "in-patient*" or camh* or "psychiatric hospital*" or " foreseeing psychiatric unit*" or "hospital patient*" or "In patient*")) AND  TOPIC: (("Delayed discharge*" or "delayed hospital discharge*" or "patient discharge*" or "blocked bed*" or "hospital stay duration*" or "discharge plan*" or "hospital patient*" or "bed avail*" or "patient transfer*" or "long term care*" or "delayed transfer of care*" or "appropriateness of stay*" or "patient discharge*" or "hospital discharge*" or "timely discharge*" or "treatment duration*" "length of stay*" or "long stay care*" or "future plan*" or "shift of care*"))  Refined by: COUNTRIES/REGIONS: ( SCOTLAND OR NORTH IRELAND OR ENGLAND OR WALES )  Timespan: All years. Indexes: SSCI. |
| **Scopus** | ( TITLE-ABS-KEY ( ( "delayed discharge*" OR "delayed hospital discharge*" OR "patient discharge*" OR "blocked bed*" OR "hospital stay duration*" OR "discharge plan*" OR "hospital patient*" OR "bed avail*" OR "patient transfer*" OR "long term care*" OR "delayed transfer of care*" OR "appropriateness of stay*" OR "patient discharge*" OR "hospital discharge*" OR "timely discharge*" OR "treatment duration*" OR "length of stay*" OR "long stay care*" OR "shift of care*" ) ) AND TITLE-ABS-KEY ( ( "long stay hospital*" OR "long stay patient*" OR "long stay unit*" OR "secure unit*" OR "medium secure unit*" OR segregate* OR "treatment facilit*" OR hospitalisation OR hospitalization OR hospital* OR "psychiatric unit*" OR "custodial institution*" OR "psychiatric secure unit*" OR "patient institutional*" OR "assessment unit*" OR "special hospital*" OR "mental health hospital*" OR "inpatient*" OR institutionalisation* OR institutionalization* OR "secure setting*" OR "secure accommodat*" OR atu* OR "assessment and treatment unit*" OR "nhs in-patient*" OR "in-patient*" OR camh* OR "psychiatric hospital*" OR "foreseeing psychiatric unit*" OR "hospital patient*" OR "in patient*" ) ) AND TITLE-ABS-KEY ( ( "learning disabilit*" OR "learning disorder*" OR "learning difficult*" OR autism OR "autistic spectrum disorder*" OR "people with learning disability*" OR "intellectual disabilit*" OR "intellectual development disorder*" OR "child and adolescent mental health*" OR "autistic spectrum*" OR "language development disorder*" OR "mental handicap*" ) ) ) AND ( EXCLUDE ( PUBYEAR , 1989 ) OR EXCLUDE ( PUBYEAR , 1988 ) OR EXCLUDE ( PUBYEAR , 1987 ) OR EXCLUDE ( PUBYEAR , 1985 ) OR EXCLUDE ( PUBYEAR , 1982 ) OR EXCLUDE ( PUBYEAR , 1979 ) OR EXCLUDE ( PUBYEAR , 1977 ) OR EXCLUDE ( PUBYEAR , 1976 ) OR EXCLUDE ( PUBYEAR , 1975 ) OR EXCLUDE ( PUBYEAR , 1974 ) OR EXCLUDE ( PUBYEAR , 1973 ) OR EXCLUDE ( PUBYEAR , 1972 ) OR EXCLUDE ( PUBYEAR , 1960 ) OR EXCLUDE ( PUBYEAR , 1946 ) ) AND ( LIMIT-TO ( AFFILCOUNTRY , "United Kingdom" ) ) |
| **Social Services Abstracts** | MAINSUBJECT.EXACT.EXPLODE("Long Term Care") OR MAINSUBJECT.EXACT.EXPLODE("Discharge") OR ti(("delayed discharge*" OR "delayed hospital discharge*" OR "patient discharge*" OR " blocked bed*" OR "hospital stay duration*" OR "discharge plan*" OR "hospital patient*" OR "bed avail*" OR "patient transfer*" OR "long term care*" OR "delayed transfer of care*" OR appropriateness of stay* " or " hospital discharge* " or " timely discharge* " or " treatment duration* " or " length of stay* " or " long stay care* " or "future plan* " or " shift of care*")) OR ab(("delayed discharge*" OR "delayed hospital discharge*" OR "patient discharge*" OR " blocked bed*" OR "hospital stay duration*" OR "discharge plan*" OR "hospital patient*" OR "bed avail*" OR "patient transfer*" OR "long term care*" OR "delayed transfer of care*" OR appropriateness of stay* " or " hospital discharge* " or " timely discharge* " or " treatment duration* " or " length of stay* " or " long stay care* " or "future plan* " or " shift of care*")) AND  [MAINSUBJECT.EXACT.EXPLODE("Segregation") OR MAINSUBJECT.EXACT.EXPLODE("Residential Institutions") OR MAINSUBJECT.EXACT.EXPLODE("Hospitalization") OR MAINSUBJECT.EXACT.EXPLODE("Mental Hospitals") OR MAINSUBJECT.EXACT.EXPLODE("Hospitals") OR ti(("Long stay hospital*" OR "Long stay patient*" OR "long stay unit*" OR "secure unit*" OR "medium secure unit*" OR segregate* OR "treatment facilit*" OR hospitali?ation OR hospital* OR "psychiatric unit*" OR "custodial institution*" OR "psychiatric secure unit*" OR "secure accommodat*" OR "patient institutional*" OR "assessment unit*" OR "special hospital*" OR "mental health hospital*" OR "inpatient*" OR institutionalisation* OR institutionalization* OR "secure setting*" OR atu* OR "assessment and treatment unit*" OR "nhs in-patient*" OR "in-patient*" OR camh* OR "psychiatric hospital*" OR "foreseeing psychiatric unit*" OR "hospital patient*" OR "In patient*")) OR ab(("Long stay hospital*" OR "Long stay patient*" OR "long stay unit*" OR "secure unit*" OR "medium secure unit*" OR segregate* OR "treatment facilit*" OR hospitali?ation OR hospital* OR "psychiatric unit*" OR "custodial institution*" OR "psychiatric secure unit*" OR "secure accommodat*" OR "patient institutional*" OR "assessment unit*" OR "special hospital*" OR "mental health hospital*" OR "inpatient*" OR institutionalisation* OR institutionalization* OR "secure setting*" OR atu* OR "assessment and treatment unit*" OR "nhs in-patient*" OR "in-patient*" OR camh* OR "psychiatric hospital*" OR "foreseeing psychiatric unit*" OR "hospital patient*" OR "In patient*"))](https://search.proquest.com/recentsearches.recentsearchtabview.recentsearchesgridview.scrolledrecentsearchlist.checkdbssearchlink:rerunsearch/EB6FDAA6750F4022PQ/None?site=socialservices&t:ac=RecentSearches) AND  [MAINSUBJECT.EXACT.EXPLODE("Learning Disabilities") OR MAINSUBJECT.EXACT.EXPLODE("Autism") OR ti(("lEarning disabilit*" OR "learning disorder*" OR "learning difficult*" OR autism OR "autistic spectrum disorder*" OR "people with learning disability*" OR "intellectual disabilit*" OR "intellectual development disorder*" OR "child and adolescent mental health*" OR "autistic spectrum*" OR "language development disorder*" OR "mental handicap*")) OR ab(("learning disabilit*" OR "learning disorder*" OR "learning difficult*" OR autism OR "autistic spectrum disorder*" OR "people with learning disability*" OR "intellectual disabilit*" OR "intellectual development disorder*" OR "child and adolescent mental health*" OR "autistic spectrum*" OR "language development disorder*" OR "mental handicap*"))](https://search.proquest.com/recentsearches.recentsearchtabview.recentsearchesgridview.scrolledrecentsearchlist.checkdbssearchlink:rerunsearch/5456D61D2E274877PQ/None?site=socialservices&t:ac=RecentSearches)  Search 2:  (ti("Learning disability hospital*" OR "intellectual disability in-patient unit*") OR ab("Learning disability hospital*" OR "intellectual disability in-patient unit*")) AND (MAINSUBJECT.EXACT.EXPLODE("Long Term Care") OR MAINSUBJECT.EXACT.EXPLODE("Discharge") OR ti(("delayed discharge*" OR "delayed hospital discharge*" OR "patient discharge*" OR " blocked bed*" OR "hospital stay duration*" OR "discharge plan*" OR "hospital patient*" OR "bed avail*" OR "patient transfer*" OR "long term care*" OR "delayed transfer of care*" OR appropriateness of stay* " or " hospital discharge* " or " timely discharge* " or " treatment duration* " or " length of stay* " or " long stay care* " or " future plan* " or " shift of care* ")")) OR ab(("delayed discharge*" OR "delayed hospital discharge*" OR "patient discharge*" OR " blocked bed*" OR "hospital stay duration*" OR "discharge plan*" OR "hospital patient*" OR "bed avail*" OR "patient transfer*" OR "long term care*" OR "delayed transfer of care*" OR appropriateness of stay* " or " hospital discharge* " or " timely discharge* " or " treatment duration* " or " length of stay* " or " long stay care* " or " future plan* " or " shift of care* ")"))) |
| **ASSIA** | (((MAINSUBJECT.EXACT.EXPLODE("Long stay patients") OR (MAINSUBJECT.EXACT.EXPLODE("Long stay") OR MAINSUBJECT.EXACT.EXPLODE("Long stay wards")) OR MAINSUBJECT.EXACT.EXPLODE("Secure units") OR MAINSUBJECT.EXACT.EXPLODE("Segregation") OR MAINSUBJECT.EXACT.EXPLODE("Secure accommodation") OR MAINSUBJECT.EXACT.EXPLODE("Hospitalization") OR MAINSUBJECT.EXACT.EXPLODE("Hospitals") OR MAINSUBJECT.EXACT.EXPLODE("Psychiatric units") OR MAINSUBJECT.EXACT.EXPLODE("Assessment centres") OR MAINSUBJECT.EXACT.EXPLODE("Special hospitals")) OR (MAINSUBJECT.EXACT.EXPLODE("Inpatient treatment units") OR ("Long stay hospital*" OR "Long stay patient*" OR "long stay unit*" OR "secure unit*" OR "medium secure unit*" OR segregate* OR "treatment facilit*" OR hospitali?ation OR hospital* OR "psychiatric unit*" OR "custodial institution*" OR "psychiatric secure unit*" OR "secure accommodat*" OR "patient institutional*" OR "assessment unit*" OR "special hospital*" OR "mental health hospital*" OR "inpatient*" OR institutionalisation* OR institutionalization* OR "secure setting*" OR "secure accomodat*" OR atu* OR "assessment and treatment unit*" OR "nhs in-patient*" OR "in-patient*" OR camh* OR "psychiatric hospital*" OR " foreseeing psychiatric unit*" OR "hospital patient*" OR "In patient*") OR ("Long stay hospital*" OR "Long stay patient*" OR "long stay unit*" OR "secure unit*" OR "medium secure unit*" OR segregate* OR "treatment facilit*" OR hospitali?ation OR hospital* OR "psychiatric unit*" OR "custodial institution*" OR "psychiatric secure unit*" OR "secure accommodat*" OR "patient institutional*" OR "assessment unit*" OR "special hospital*" OR "mental health hospital*" OR "inpatient*" OR institutionalisation* OR institutionalization* OR "secure setting*" OR "secure accomodat*" OR atu* OR "assessment and treatment unit*" OR "nhs in-patient*" OR "in-patient*" OR camh* OR "psychiatric hospital*" OR " foreseeing psychiatric unit*" OR "hospital patient*" OR "In patient*")))  AND (MAINSUBJECT.EXACT.EXPLODE("Community based discharge planning") OR MAINSUBJECT.EXACT.EXPLODE("Discharge planning") OR MAINSUBJECT.EXACT.EXPLODE("Bed blocking") OR MAINSUBJECT.EXACT.EXPLODE("Long term care") OR MAINSUBJECT.EXACT.EXPLODE("Long stay patients") OR MAINSUBJECT.EXACT.EXPLODE("Hospital discharged") OR MAINSUBJECT.EXACT.EXPLODE("Long stay wards") OR ("Delayed discharge*" OR "delayed hospital discharge*" OR "patient discharge*" OR "blocked bed*" OR "hospital stay duration*" OR "discharge plan*" OR "hospital patient*" OR "bed avail*" OR "patient transfer*" OR "long term care*" OR "delayed transfer of care*" OR "appropriateness of stay*" OR "patient discharge*" OR "hospital discharge*" OR "timely discharge*" OR "treatment duration*" "length of stay*" OR "long stay care*" OR "future plan*" OR "shift of care*") OR ("Delayed discharge*" OR "delayed hospital discharge*" OR "patient discharge*" OR "blocked bed*" OR "hospital stay duration*" OR "discharge plan*" OR "hospital patient*" OR "bed avail*" OR "patient transfer*" OR "long term care*" OR "delayed transfer of care*" OR "appropriateness of stay*" OR "patient discharge*" OR "hospital discharge*" OR "timely discharge*" OR "treatment duration*" "length of stay*" OR "long stay care*" OR "future plan*" OR "shift of care*"))  AND (MAINSUBJECT.EXACT.EXPLODE("Learning disabilities") OR MAINSUBJECT.EXACT.EXPLODE("Autism") OR MAINSUBJECT.EXACT.EXPLODE("Autistic spectrum disorders") OR ("learning disabilit*" OR "learning disorder*" OR "learning difficult*" OR autism OR "autistic spectrum disorder*" OR "people with learning disability*" OR "intellectual disabilit*" OR "intellectual development disorder*" OR "child & adolescent mental health" OR "autistic spectrum*" OR "language development disorder*" OR "mental handicap") OR ("learning disabilit*" OR "learning disorder*" OR "learning difficult*" OR autism OR "autistic spectrum disorder*" OR "people with learning disability*" OR "intellectual disabilit*" OR "intellectual development disorder*" OR "child & adolescent mental health" OR "autistic spectrum*" OR "language development disorder*" OR "mental handicap")) AND (loc.exact("United Kingdom--UK" OR "England" OR "Wales" OR "Scotland" OR "UK" OR "United Kingdom") AND yr(1990-2029)))  OR ((ti("Learning disability hospital*" OR "intellectual disability in-patient unit*") OR ab("Learning disability hospital*" OR "intellectual disability in-patient unit*")) AND (MAINSUBJECT.EXACT.EXPLODE("Community based discharge planning") OR MAINSUBJECT.EXACT.EXPLODE("Discharge planning") OR MAINSUBJECT.EXACT.EXPLODE("Bed blocking") OR MAINSUBJECT.EXACT.EXPLODE("Long term care") OR MAINSUBJECT.EXACT.EXPLODE("Long stay patients") OR MAINSUBJECT.EXACT.EXPLODE("Hospital discharged") OR MAINSUBJECT.EXACT.EXPLODE("Long stay wards") OR ("Delayed discharge*" OR "delayed hospital discharge*" OR "patient discharge*" OR "blocked bed*" OR "hospital stay duration*" OR "discharge plan*" OR "hospital patient*" OR "bed avail*" OR "patient transfer*" OR "long term care*" OR "delayed transfer of care*" OR "appropriateness of stay*" OR "patient discharge*" OR "hospital discharge*" OR "timely discharge*" OR "treatment duration*" "length of stay*" OR "long stay care*" OR "future plan*" OR "shift of care*") OR ("Delayed discharge*" OR "delayed hospital discharge*" OR "patient discharge*" OR "blocked bed*" OR "hospital stay duration*" OR "discharge plan*" OR "hospital patient*" OR "bed avail*" OR "patient transfer*" OR "long term care*" OR "delayed transfer of care*" OR "appropriateness of stay*" OR "patient discharge*" OR "hospital discharge*" OR "timely discharge*" OR "treatment duration*" "length of stay*" OR "long stay care*" OR "future plan*" OR "shift of care*"))) |
