# Supplementary material for: ‘Why are we stuck in hospital?’ Understanding delayed hospital discharges for people with learning disabilities and/or autistic people in long‐stay hospitals in the UK
Source: Health Soc Care Community. 2022 Aug 11;30(6):e3477–92. doi: 10.1111/hsc.13964 (PMC10087420; doi:10.1111/hsc.13964)
Supplement: Supplementary file 2 — Appendix S2 [file HSC-30-e3477-s001.docx]

Appendix B

Search results from selected databases

| **Title of database and dates covered** | **Name of the database provider / host (e.g. OVID)** | **Date search conducted and dates covered by the search (e.g. 1990- February week 3, 2016)** | **Results** |
| --- | --- | --- | --- |
| **ASSIA** | Proquest | 09/02/2021 | 236 |
| **HMIC** | Ovid | 08/02/2021 | 113 |
| **Medline** | Ovid | 08/02/2021 | 64 |
| **Scopus** | Elsevier | 09/02/2021 | 396 |
| **Social Policy & Practice (incorporating Social Care Online, Ageinfo and Caredata)** | Ovid | 08/02/2021 | 59 |
| **SSCI** | Thomson Reuters Web of Science | 08/02/2021 | 27 |
| **Social Services Abstracts** | Proquest | 09/02/2021 | 2 |
| Results |  |  | **897** |
| FINAL SEARCH RESULTS (after de-duplication) |  |  | **785** |
